# Extent of Mangrove Nursery Habitats Determines the Geographic Distribution of a Coral Reef Fish in a South-Pacific Archipelago

**DOI:** 10.1371/journal.pone.0105158

**Published:** 2014-08-20

**Authors:** Christelle Paillon, Laurent Wantiez, Michel Kulbicki, Maylis Labonne, Laurent Vigliola

**Affiliations:** 1 Institut de Recherche pour le Développement (IRD), UR227 CoRéUs, Laboratoire Excellence LABEX corail, Nouméa, New Caledonia; 2 Université de Nouvelle-Calédonie (UNC), LIVE (EA4243), Nouméa, New Caledonia; 3 Institut de Recherche pour le Développement (IRD), UR227 CoRéUs, Laboratoire Excellence LABEX corail, c/o Laboratoire Arago, Banyuls sur Mer, France; 4 Institut de Recherche pour le Développement (IRD), LEMAR (UMR 6539), Institut Universitaire Européen de la Mer, Plouzané, France; University of Otago, New Zealand

## Abstract

Understanding the drivers of species' geographic distribution has fundamental implications for the management of biodiversity. For coral reef fishes, mangroves have long been recognized as important nursery habitats sustaining biodiversity in the Western Atlantic but there is still debate about their role in the Indo-Pacific. Here, we combined LA-ICP-MS otolith microchemistry, underwater visual censuses (UVC) and mangrove cartography to estimate the importance of mangroves for the Indo-Pacific coral reef fish *Lutjanus fulviflamma* in the archipelago of New Caledonia. Otolith elemental compositions allowed high discrimination of mangroves and reefs with 83.8% and 98.7% correct classification, respectively. Reefs were characterized by higher concentrations of Rb and Sr and mangroves by higher concentrations of Ba, Cr, Mn and Sn. All adult *L. fulviflamma* collected on reefs presented a mangrove signature during their juvenile stage with 85% inhabiting mangrove for their entire juvenile life (about 1 year). The analysis of 2942 UVC revealed that the species was absent from isolated islands of the New Caledonian archipelago where mangroves were absent. Furthermore, strong positive correlations existed between the abundance of *L. fulviflamma* and the area of mangrove (r = 0.84 for occurrence, 0.93 for density and 0.89 for biomass). These results indicate that mangrove forest is an obligatory juvenile habitat for *L. fulviflamma* in New Caledonia and emphasize the potential importance of mangroves for Indo-Pacific coral reef fishes.

## Introduction

Coral reefs and mangroves are amongst the most productive ecosystems of the world and have high economic values [1]. They are also highly impacted by human activity, with 30% of coral reefs already severely impacted and a 50% decrease of mangrove area in the last 50 years [2]–[4]. Natural disturbances in conjunction with increasing human pressures have led to dramatic losses of quality and extent of these ecosystems [5]–[10]. Coral reef seascapes are fragmented ecosystems where local populations of reef organisms are connected by movements of individuals at the pelagic larval, and benthic juvenile/adult stages [11]. After a pelagic life of variable duration, larvae settle in shallow nursery habitats such as mangroves, seagrass beds or reefs [12] where juveniles may benefit from several advantages such as food availability and shelter from predators [13]. As they grow, individuals move to coral reefs and recruit into adult populations [14].

Mangrove forests are habitat for many juvenile coral reef fish species (e.g. Serranidae, Lutjanidae, Lethrinidae, etc.) as they provide shelter and food and therefore increase the overall rate of survival for juveniles [13], [15]–[17]. As a result, there can be strong relationships between fish abundance and the presence of mangroves with, for example, greater biomass of Lutjanidae in reefs close to mangroves in the Caribbean [4], [18], [19]. However, some species using mangroves during their juvenile life stage can also be observed in high density in other juvenile habitats (e.g. seagrass beds, soft bottoms, fringing reefs) [14] and the absence of mangroves may have no significant impact on adult fish population densities on nearby reefs [19]. Thus, the importance of mangroves as nurseries may vary depending on the species and the region. As defined by Adams *et al.* [20], mangroves can be obligatory, important or facultative juvenile habitats. They are obligatory when species cannot complete their life cycle in the absence of mangroves. They are important when they make large contributions to adult populations. Finally, they are facultative nursery habitats when the species is abundant in numerous habitats as juveniles, and thus only a minority of juveniles is found in mangroves [20]. Following these three possibilities, the relationship between the abundance of the species and the extent of mangroves will be different.

The connectivity between mangroves and coral reefs for ontogenetic shifters suggests that the distribution of juvenile habitats may be a primary determinant of species' distribution when the juvenile habitat is an obligatory one. It has often been assumed that larval transport is the primary determinant of population connectivity and geographic range size. However, in a recent paper Luiz *et al*. [21] showed that adult traits were equal or better predictors of geographic range size than larval traits, highlighting the importance of post-settlement processes likely to affect the capacity of new colonizers to survive and establish reproductive populations. Testing whether the distribution of a juvenile habitat (e.g. mangrove) is a primary determinant of a species' distribution requires assessing if this habitat is obligatory, important or facultative. This implies the need to estimate the proportion of adults that effectively used the juvenile habitat as nursery. Methods used to directly measure connectivity between juvenile and adult populations require natural or artificial habitat-specific markers [20], [22], [23]. Among these methods, the microchemistry of otoliths is one of the most powerful tools to identify juvenile habitats when adult and juvenile populations are spatially distinct [16], [24]–[28]. Otoliths are paired calcified structures located in the inner ear of fishes. They show continuous growth allowing an incorporation of trace elements during the entire lifetime of a fish. Due to their inorganic nature, trace elements are not resorbed after incorporation. These key properties allow the use of otoliths as natural recorders of the habitats inhabited by fish throughout their lives [29]. In particular, it is possible to analyze the chemical composition of the juvenile part of the otolith of an adult and retrospectively determine the habitats used by this adult when it was a juvenile.

The purposes of this study were to: 1) investigate whether, in New Caledonia, mangroves are obligatory, important or facultative juvenile habitats for *Lutjanus fulviflamma* (Forsskal, 1775) (Lutjanidae) and 2) test whether the adult distribution of this species is related to the geographic distribution of mangroves in the New Caledonia archipelago, using otolith microchemistry to characterize the chemical signature of habitats and an underwater visual census (UVC) database (2942 transects) to determine the relationship between the abundance of this species and the mangroves.

## Materials and Methods

### Sampling protocol

**Figure 1:**
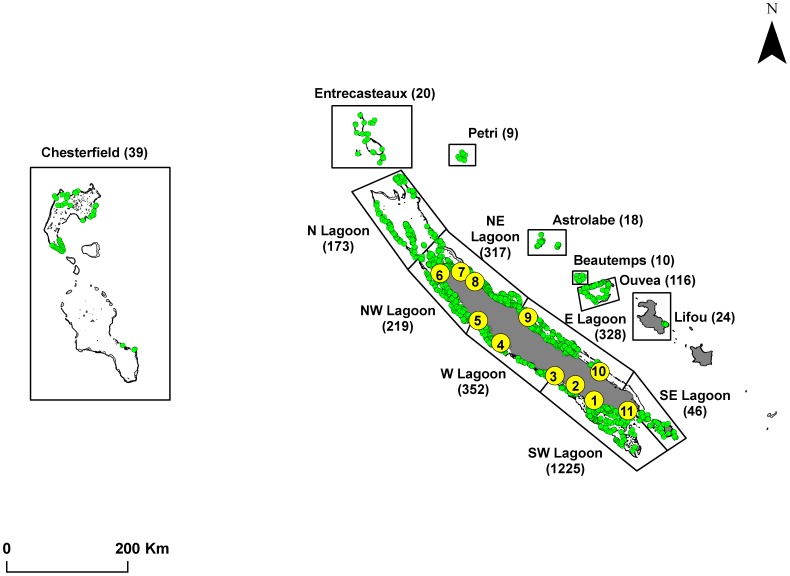
Map of New Caledonia with sampling sites for microchemistry (yellow circles) and underwater visual censuses (UVC, green circles). Polygons indicate the different lagoons, remote reefs and islands for which UVC data were collected; numbers indicate the number of UVC transects.

New Caledonia is an archipelago located in the South West Pacific, 1500 km east of Australia, between latitudes 19°S and 23°S and longitudes 163°E and 168°E (Fig. 1). It is composed of a main island (Grande Terre), three groups of smaller islands (Loyalties, Isle of Pines and Belep) and some uninhabited islands and offshore reefs (Entrecasteaux, Chesterfield, Astrolabe, Petri, Matthew, Hunter and Walpole). The main island holds the largest lagoon of the world, with a linear barrier reef of 1744 km long and a surface area of 31 336 km^2^ [30]. Distance between the main island and the barrier reef varies from 1 to 65 km [31] and major parts of the lagoon are registered on the Unesco heritage list.

*Lutjanus fulviflamma* (Forsskål, 1775) or blackspot snapper (Family Lutjanidae) is a small Indo-Pacific snapper commonly targeted by subsistence fisheries. It is a carnivorous species with a mean adult size of 25 cm, found in different habitats according to its ontogenetic stage. Adults inhabit coral reefs between 3 to 35 m deep and juveniles are known to use shallow-waters habitats like mangrove forests [32]–[34].

Fish were sampled for otolith analysis in different mangrove and reef habitats at 11 sites around the main land of New Caledonia over three years (2009, 2010 and 2011) (Fig. 1, Table S1). Adults were collected in reef habitats by spear fishing and juveniles were collected in mangroves using small mesh (7 mm) gillnets surrounding an isolated mangrove tree. Gillnets were deployed at high tide and fish collected with handnets in a few cm of water and/or on the ground at low tide. Immediately after collection, fish were put on iced water (<5°C) to be anesthetized. Afterwards, they were transported to the laboratory where they were stored in a freezer (at −20°C) until dissection.

The first sampling took place during austral summer 2009 in the mangroves and inner barrier reefs of two sites (Gatope and St Vincent). The second sampling was carried out during austral winter 2010 in the mangroves and inner barrier reefs of eleven sites around the main island (Fig. 1). During this sampling, fringing and intermediate reefs were also sampled at four sites (Gatope, Ouano, Prony and St Vincent). Sampling in the mangroves, fringing, intermediate and inner barrier reefs at these 4 sites was repeated during austral winter 2011 (Table 1). Thus, our sampling included fish collected in different years, seasons and habitats, which is important as chemical signatures recorded in fish otoliths may vary over space and time, and spatio-temporal variability needs to be included in the characterization for robust chemical signatures in otoliths [35].

**Table 1:** Sampling design and chemical analyses.

Objectives	Year	Number of sites	Habitat	Laser analysis	*n* otoliths
A. Characterization of habitat signatures	2009	2	M, BR	Ablation on surface	20
	2010	7	M, BR	Ablation on surface	51
	2010	4	M, FR, IR, BR	Ablation on surface	63
	2011	4	M, FR, IR, BR	Ablation on surface	62
B. Determination of juvenile habitats	2010	4	BR	Transects	20

Sampling design and chemical analyses to: (A) characterize reefs and mangrove chemical signatures by LA-ICPMS of the surface of otoliths and (B) determine the juvenile habitat by LA-ICPMS of transverse sections of otolith along transects from core to 1260 µm; M: Mangrove; FR: Fringing Reef; IR: Intermediate Reef; BR: Barrier Reef.

Ethics and sampling approvals were provided by the Environmental Department of the Southern Province of New Caledonia for Nouméa, St Vincent, Ouano, Port Bouquet and Prony sites (permit #1103-2010/ARR/DENV), and by the Economic Development and Environmental Department of the Northern Province of New Caledonia for Népoui, Gatope, Golonne, Amos, Tchambouenne and Paama sites (permit #60912-2010/JJC). In this study, no protected species was collected and collections were not made in protected areas.

### Otolith preparation

All equipment used for otolith handling was previously decontaminated in 5% ultrapure nitric acid bath during 24 h, rinsed three times with ultrapure water (18.2 MΩ), dried under a laminar flow hood (HEPA 100) and stored dry in plastic bag under the flow hood. At the lab, fish samples were unfrozen and each individual was measured (Fork length, FL) to the nearest mm and weighted to the nearest gram. Paired sagittal otoliths were extracted using ceramic tweezers, cleaned with ultrapure water (18.2 MΩ) and stored dry in plastic vials.

The chemical signatures of reefs and mangroves were characterized by analyzing the chemical composition at the surface of otoliths (outer edge) of up to five fish randomly selected in each sample (Table 1A). This method proposed by Warner *et al.* [38] allows characterizing the latest elemental signature recorded in the otolith, which corresponds to the habitat where the fish was caught [35]–[37]. Otoliths of these fishes were cleaned of organic material following Warner *et al.* 's [38] method: otoliths were bathed in a 50/50 H_2_O_2_ (30%, Suprapur grade) and NaOH (Suprapur grade, 0.1 mol.L^−1^) solution during 1 hour, sonicated during the last 5 min of the bath, rinsed 5 times with ultrapure water for 5 min, dried under a laminar flow hood (HEPA 100) and stored dry in individual vials until microchemistry analyses.

The juvenile habitats used by adults were inferred from the analyses of the chemical composition along transverse otolith sections of 20 adult fish collected on the barrier reefs of Gatope, Ouano, Prony and St Vincent (5 fish per site, Table 1B). Transverse otolith sections including otolith cores were prepared by embedding right sagittal otoliths in epoxy resin, sectioning transversely with a low speed saw, and polishing using sandpaper of decreasing grain (from 800 to 1600 grains/cm^2^), lapping film (9 to 1 µm) and diamond-tipped polishing powders. Finally, sections were rinsed in ultrapure water, dried under a laminar flow hood (HEPA 100), mounted on a microscope slide (10 sections per slide) with double-side tape and stored in plastic boxes until microchemistry analyses.

### Otoliths LA-ICP-MS analyses

Otoliths were chemically analyzed by Laser Ablation Inductively Coupled Plasma Mass Spectrometry (LA-ICP-MS) at the Pôle de Spectrométrie Océan (Institut Européen Universitaire de la Mer, Brest, France) using a Thermo Element 2 coupled to a laser 193 nm CopexPro 102 Coherent, and at the Research Institute of Environment and Livelihoods (Charles Darwin University, Darwin, Australia) using an Agilent 7500ce coupled to a UP – 213 nm laser ablation system. Both ICP-MS were operated at low resolution using argon as the carrier gas, and with the laser system parameters set on a 90 µm laser beam diameter and a frequency of 5 Hz. Each analysis lasted 120 s including 30 s of background and 90 s of ablation (laser activated). To discriminate habitats, the following isotopes were measured: ^7^Li, ^11^B, ^85^Rb, ^88^Sr, ^95^Mo, ^111^Cd, ^117^Sn, ^138^Ba, ^208^Pb, ^232^Th, ^238^U, ^25^Mg, ^43^Ca, ^47^Ti,^ 51^V, ^52^Cr, ^55^Mn, ^60^Ni, ^65^Cu and ^66^Zn.

To measure the microchemistry signature of the habitat where the fish was caught, the surface of the otolith of each subsampled fish (up to 5 individuals per sample) was laser ablated following Warner *et al.* [38]. To standardize the analyses, the ablation was always done at the same spot at the tip of the post rostrum on the posterior side of otoliths.

To determine the juvenile habitat of adults, the juvenile parts of otolith transverse sections were analyzed by LA-ICPMS for each of the 20 adult fish collected in the barrier reefs of Gatope, Ouano, Prony and St Vincent. Because the otolith radius of the smallest juvenile caught in our samples was 1260 µm, we considered this radius as the juvenile part of otolith sections. For each adult, one LA-ICPMS transect was analysed. It consisted of ten successive laser ablations (point-by-point mode) from the core to 1260 µm by increments of 140 µm along the longest otolith growth axis.

To compensate possible variations due to differences in quantity of material ablated, calcium was used as an internal standard. An external standard (National Institute of Standards and Technology, NIST, 612) was analyzed twice at the beginning and at the end of each session (8 otoliths per session) and also after ten ablations for each transect in order to correct for instrument drift.

Raw ICPMS data (counts per second, CPS) were first cleaned of outliers, defined as any value greater than three times the inter-quartile distance, during both the blank and the ablation windows [39]. Cleaned CPS were then transformed in elemental concentrations (parts per million, ppm) and limits of detection (LOD) calculated following Longerich *et al* [40]. Only the elements that met the following two criteria were kept in statistical analyses: 1) elemental concentrations had to be greater than the LOD in 70% of the otoliths in at least one habitat (e.g. mangrove) or one site (e.g. Ouano) and 2) the coefficient of variation of concentrations in the NIST 612 had to be less than 10% [23], [41]. When the concentration of an element was under the LOD, it was set to zero. By definition, this occurred in less than 30% of the cases in at least one habitat or one site (otherwise the element was removed from our analyses based on criterion 1). To reduce variation originating from analyses produced by two different ICP-MS, elemental concentrations were transformed to percent of all measured elements.

In order to correlate juvenile habitat use with age and size, the 20 adults were aged by counting the number of yearly increments (opaque bands) on otoliths and size were back-calculated from measurement of otolith radius at each ablation spot along the transects using the Dahl-Lea back-calculation model [42]: *L_i_* =  (*R_i_*/*R_cpt_*) ×*L_cpt_* where *R_i_* and *L_i_* are otolith radius and fish length at ablation spot *i*, *R_cpt_* and *L_cpt_* are otolith radius and fish length at capture. We assumed that one annulus was formed each year as it is the case for several Lutjanidae species closed to the study specie [43], [44].

### UVC surveys and mangrove area calculation

The fish diversity on the coral reefs of New Caledonia has been surveyed by IRD and UNC since 1986. Over the years, we built a database containing 2942 Distance-sampling Underwater Visual Census (D-UVC). The method consists of recording species name, abundance, body length and distance to transect line for each fish or group of fish observed along a 50 m transect. The distance-sampling method is fully described in Labrosse *et al.* [45]. The presence/absence of *L. fulviflamma* was directly extracted from our raw database and abundance calculated after truncation of original D-UVC datasets at a distance of 5 m on each side of the transect line [46], effectively rendering D-UVC transects equivalents to 500 m^2^ belt transects (50 m long ×5 m wide ×2 sides). Density of *L. fulviflamma* was therefore calculated by dividing the number of fish counted within the truncated D-UVC transects by 500 m^2^. Biomass was calculated from density and length-weight coefficients [47]. All transects were layed parallel to the reef between 0 to 15 meters depth and encompassed the full range of habitats presents in New Caledonia.

The presence, frequency of occurrence, average density and biomass of *L. fulviflamma* were calculated for the different lagoons of New Caledonia (as defined by Pelletier [48]) and the remote reefs and islands of the archipelago for which we had data (Fig. 1). Surface area of mangroves standardized by coast length (to account for the difference in size of the different lagoons) were calculated for the same lagoons, reefs and islands using the mangrove cartography of Virly [49] available at www.georep.nc.

### Data analyses

One-way PERMANOVA (with 999 permutations) based on Euclidian distance [50], [51] was performed to test the effect of habitat (mangrove versus reef) on the multi-elemental composition of otoliths. This test was followed by one-way PERMANOVAs with 999 permutations in order to compare the chemical concentrations of each element between mangroves and reefs.

The random forests (RF) algorithm [52] was performed on elemental concentrations from otolith surfaces to build an habitat classifier based on otolith microchemistry. This classification method allows freedom from normality and homoscedasticity assumptions and is the most appropriate for otolith microchemistry data [35], [53]. With the RF classification method, 75% of the dataset are used to build a classification tree. Then, the remaining 25% are classified along this tree. The RF algorithm built 5000 trees to ensure that every individual gets predicted several times, which permits to estimate classification accuracy (percent of correct classification). Furthermore, we performed RF for all possible element combinations in order to find the combination that showed the greatest classification accuracy. The RF classifier based on this best combination of elements was then used to predict the juvenile habitats corresponding to the chemical composition of the different LA-ICPMS analyses performed along the otolith transverse sections of the 20 adults collected on the barrier reefs of Gatope, Ouano, Prony and St Vincent. A PCA was also used to further visualize the elemental composition of otoliths from fish collected in different habitats. Elemental compositions were *arcsin√x* transformed in the PCA to normalize the data.

The relationship between the presence of mangroves and *L. fulviflamma* in the different lagoons, remote reefs and islands of the Archipelago of New Caledonia was tested by a Chi-square test. Spearman rank correlations were calculated to test the relationship between mangrove and fish abundance (frequency of occurrence, density, and biomass).

## Results

### Building a RF habitat classifier from otolith microchemistry

Twelve elements met the criteria of selection and were retained for statistical analyses: B, Ba, Cr, Mg, Mn, Sn, Sr, Rb, U and Zn. A PERMANOVA showed that the overall elemental composition of otoliths was significantly different between mangrove and reef habitats (F_1,194_ = 31.67; p<0.01). One-way PERMANOVA further revealed significant differences between habitats for six elements: Ba, Cr, Mn, Sn, Sr and Rb (Fig. S1). Elemental concentrations of Ba, Cr, Mn and Sn were significantly greater in otoliths of individuals collected in mangroves than those collected in reefs. On the contrary, Rb and Sr were at lower concentrations in otoliths from mangroves than in reef otoliths.

The best Random Forest (RF) classification was obtained with a combination of only three elements among the twelve tested: Mn, Sn and Rb. With these three elements, the RF was able to correctly classify 95.9% of the fish in the habitat where they were collected. However, percentages of correct classification varied with the habitats. 83.3% of the fish from mangroves were correctly classified in their sampling habitat and 98.7% of the fish from reefs were correctly classified (Table 2).

**Table 2:** Classification matrix of habitats obtained from Random Forest analyses.

Habitats	Correct classification (%)	Number of fish classified in		
		Mangrove	Reefs	Total
Mangrove	83.8	31	6	37
Reefs	98.7	2	157	159
Total	95.9	33	163	196

**Figure 2:**
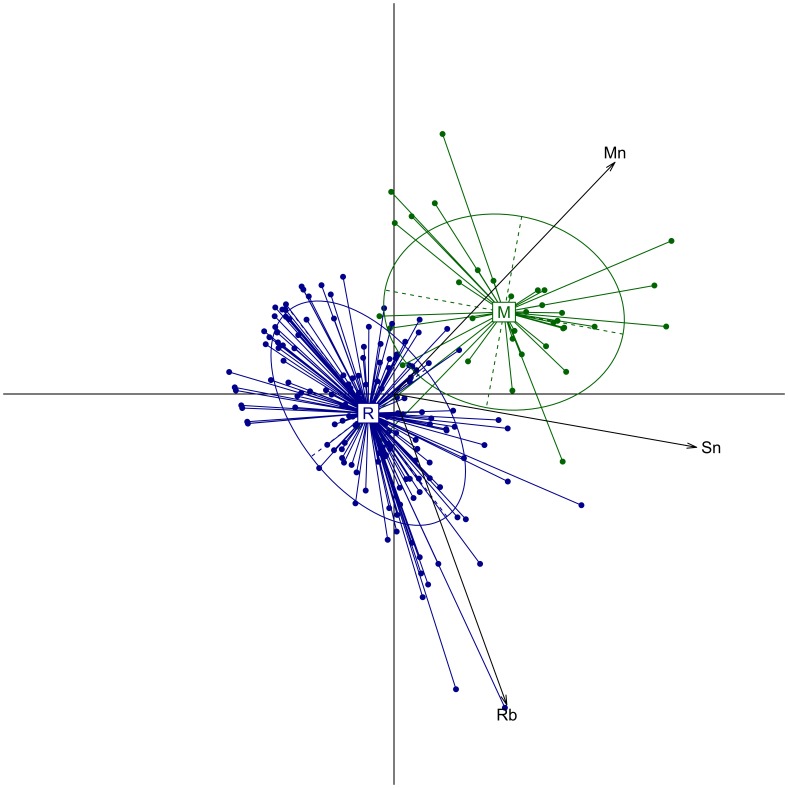
PCA biplot of otolith elemental concentrations from mangroves and reefs. Green and blue dots indicate the 37 fishes collected in mangroves (M) and the 159 fishes collected in reefs (R), respectively. Only concentrations of Rb, Sn and Mn in otoliths were used in the PCA.

A PCA plot further showed the role of manganese (Mn), rubidium (Rb) and tin (Sn) in the discrimination of mangrove from the reef habitats with Mn and Sn characterizing mangroves whereas Rb was characteristic of reefs (Fig. 2).

### Back-calculating juvenile habitats of adults

**Figure 3:**
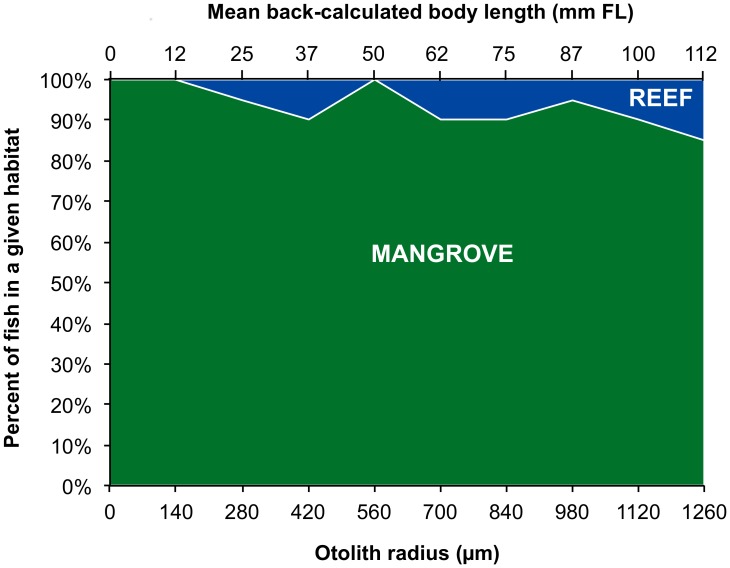
Proportion of adults *L. fulviflamma* occupying mangroves as juvenile. Results of RF prediction of habitats obtained along the juvenile part of otolith transects of the 20 adults *L. fulviflamma* collected in barrier reefs. For each fish and at each laser ablation along otolith transects, RF predict the habitat (mangrove or reef) corresponding to the chemical analysis. The green area represents the proportion of fish with a mangrove signature whereas the blue area represents the proportion of fish with a reef signature. Mean fish body length back-calculated from otolith radius are given in upper-x axis.

Applying the RF best classifier to all the chemical analyses done from the core to the distance of 1260 µm showed that all 20 adult *L. fulviflamma* collected on barrier reefs had a mangrove signature on their two first laser ablations (0 and 140 µm) (Fig. 3). A mangrove signature was predicted for 85% of fishes (17 individuals) from 280 µm to the last analysis at 1260 µm. According to the classifier, only one fish collected at the site of Prony used mangrove as juvenile habitat during a very short period of time. The 20 analyzed fish ranged from 4 and to 18 years in age and from 18 to 28 cm in fork length. At an otolith radius of 1260 µm, 16 fish had one year, 4 had less than one year, and back-calculated body length ranged from 96 to 128 mm fork length (mean 112 mm ±9 SD, n = 20).

### Relationships between mangrove and fish abundance

**Figure 4:**
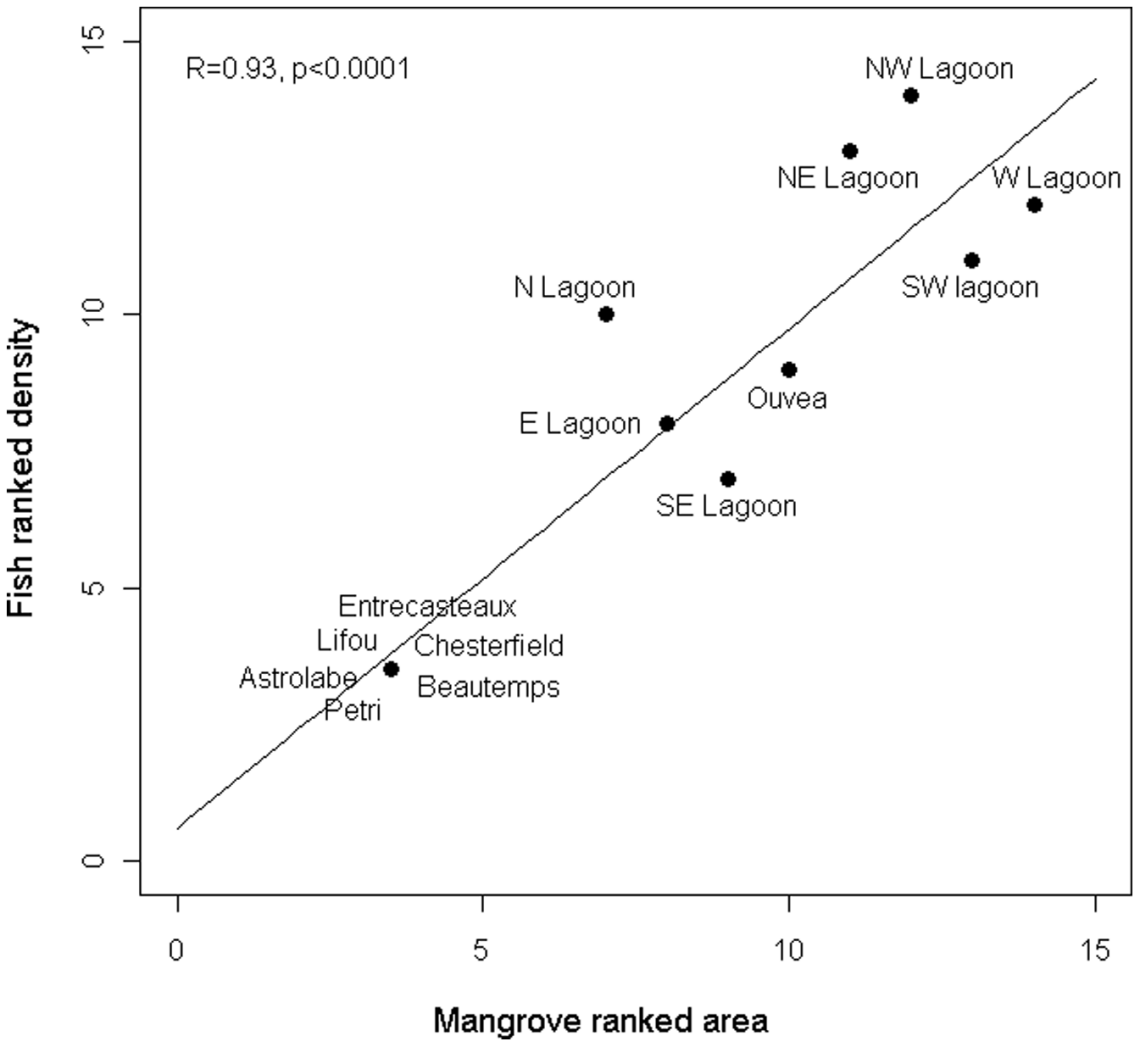
Relationship between *L. fulviflamma* density and mangrove area. Spearman-rank correlation between standardized mangrove area and density of *L. fulviflamma* in the different lagoons, reefs and islands of the archipelago of New Caledonia (see map on Fig. 1) is given.

UVC surveys showed that the species was not recorded in remote islands where mangroves are absent (Chesterfield, Entrecasteaux, Petri, Astrolabe, Beautemps-Beaupré, Lifou) and was always recorded in places where mangroves are present (Table S2). Interestingly, some *L. fulviflamma* were recorded in the atoll of Ouvéa where mangroves are present but not in the nearby atoll of Beautemps-Beaupré where mangroves are absent. The strong relationship between the presence of mangroves and the presence of *L. fulviflamma* was confirmed by a highly significant chi-square test (χ^2^ = 14, df = 1, P<0.0002). Spearman rank correlations showed highly significant relationships between standardized mangrove areas and the frequency of occurrence (R = 0.84, P<0.0002), density (R = 0.93, P<0.0001) and biomass (R = 0.89, P<0.0001) of *L. fulviflamma*. Fish abundance was 1) greater along the West coast of the mainland and in the NE lagoon where mangroves are well developed, 2) lower in the North Lagoon where reefs are very far away from the mainland, and in the East, South East lagoons and Ouvéa island where mangrove are scarce, and 3) absent in the reefs, atolls and islands disconnected from the mainland and where mangroves are absent (Fig. 4).

## Discussion

The geographic distribution of species is a fundamental ecological parameter because it strongly influences species' susceptibility to extinction. As such, understanding the drivers of species' geographic distributions is crucial for the development of strategic conservation plans. Up to now, it was generally believed that the main determinant of geographic range size of marine organisms was larval dispersion and a few studies have indeed reported a positive relationship between range size and planktonic larval duration [54]–[56]. However, many other studies found no relationship [57], [58]. Very recently, Luiz *et al*. [21] revised the larval dispersal paradigm and reported that adult traits were as important as larval traits in explaining the geographic range size of reef fish species. To explain their findings, they proposed the following hypothesis: to found a new population, new colonizers not only must arrive on a new habitat as settling larvae, but they must also survive and find a mate to reproduce. And indeed, many adult traits such as large size or schooling behavior may diminish predation risks and consequently increase the probability of new colonizers to survive and establish new populations. If post-settlement survival is a crucial determinant of a species' geographic range size, then we hypothesized that the spatial distribution of those habitats which are obligatory for the survival of juveniles should closely match the spatial distribution of the species. In order to test this hypothesis, we selected a reef fish species known to use mangroves as nurseries, applied otolith microchemistry techniques to determine whether mangroves were obligatory, important or facultative juvenile habitats, and explored the relationships between species abundance and mangrove extent in the archipelago of New Caledonia.

### Characterization of juvenile habitat

The first objective of this study was to identify the role of mangroves as juvenile habitat for the blackspot snapper *Lutjanus fulviflamma* in New Caledonia using otolith microchemistry. We found that the otolith chemical composition of *Lutjanus fulviflamma* allowed almost perfect discrimination of mangrove from reef ecosystems using only three elements (Mn, Sn and Rb), and that 6 elements varied significantly between the two ecosystems. Ba, Cr, Mn and Sn were in greater concentrations in otoliths of fish from mangroves whereas Rb and Sr were in greater concentrations in otoliths of fish from coral reefs. In agreement with our study, Chittaro *et al.* [23] reported Sr concentrations significantly greater in otoliths from reefs than mangroves in the Bahamas.

The power of otolith microchemistry to identify juvenile and adult habitats resides in the distinct chemical composition of otoliths collected from different habitats [59]. Mangroves grow in coastal areas often showing chemical heterogeneity due to different inputs (anthropogenic, fluvial and upwelling) compared with more oceanic coral reef environments. In addition, New Caledonia shows a peculiar geological context and a significant past and present mining activity with past exploitations of Mn and Cr on the Main Island [31]. Moreover, Mn shows a scavenging profile type, which means higher concentrations in coastal waters and a tendency to decrease with distance from the coastline [60]. Higher levels of Mn and Sn in mangroves could also originate from fertilizers used for agriculture and could be higher near areas with freshwater inputs [61]. Furthermore, Sn was present in antifouling paints until late 2012 in New Caledonia, which could also have contaminated coastal waters near harbors. Finally, differences in Ba and Sr concentrations between reefs and mangroves may be explained by gradients in salinity and temperature between the two habitats [59].

### Essentialness of mangrove

The second objective of our study was to determine whether mangroves were obligatory, important or facultative juvenile habitats for *L. fulviflamma* in New Caledonia. To achieve this goal, we looked for elemental signatures of mangroves in the juvenile parts of otoliths from adults collected on barrier reefs. The results showed that all analyzed fish had a mangrove signature at the beginning of their life.

Coastal shallow-water habitats are recognized worldwide as important nursery areas for fish [13], [15], [20]. Many studies, mostly conducted in the Caribbean region, established the nursery role of mangroves and their attractiveness for juveniles [4], [15]–[17], [19], [62]–[64]. Our study took place in the South Pacific region where there is a lack of knowledge on mangrove-reef interactions [20]. In New Caledonia, previous studies showed that mangroves were used by blackspot snappers as one juvenile habitat among other shallow-water habitats [32], [34], [65]. Our study used otolith microchemistry, a powerful method to study the role of mangroves as nurseries [23], [41], [66], [67] and revealed the true importance of mangroves for *L. fulviflamma*: all analyzed adults lived in mangroves as juveniles, which suggests that mangroves are probably an obligatory juvenile habitat for this species in New Caledonia.

### Geographic distribution

Although a sample size of 20 adults is probably not sufficient to affirm with certainty that mangroves are indeed an obligatory juvenile habitat, the relationship that we found between the presence of mangroves and the presence of the species strongly supports our conclusion from otolith microchemistry. New Caledonia is an archipelago with a large main island colonized by mangroves and surrounded by a complex coral reef ecosystem, and several islands and offshore reefs with or without mangroves. *L. fulviflamma* was present all around the mainland, including on reefs very far away from the main land, suggesting that individuals who completed their juvenile life in the mangroves of the mainland were able to swim long distances within an interconnected reef ecosystem. Furthermore, the species was present in offshore islands where mangroves are present, suggesting that planktonic larvae transported by oceanic currents can reach these islands and survive as juveniles in the mangroves. However, the species was not present at d'Entrecasteaux atolls, Astrolabe reefs, Chesterfield banks, Pétri atoll, Beautemps-Beaupré atoll and Lifou Island. One hypothesis to explain this absence is a lack of connectivity at the larval stage. However, this hypothesis is unlikely because models of larval dispersion indicate persistent connectivity among reefs in the archipelago of New Caledonia for species with pelagic larval duration (PLD) greater than 15 days [68], and *L. fulviflamma* has an average PLD of 23 days [21]. Furthermore, it would be very surprising that larvae can reach the atoll of Ouvéa where the species and mangrove are present and not the nearby atoll of Beautemps-Beaupré or Astrolabe reef where the species and mangrove are absent. Likewise, the hypothesis of overfishing seems unlikely because some of the sites where the species was absent are strictly protected while, on the contrary, the species was present at sites with the highest fishing pressure, like Nouméa, the capital city of New Caledonia. Clearly, the most plausible hypothesis for the absence of the species at these reefs and islands is the absence of mangrove, a probable obligatory juvenile habitat for this species in New Caledonia.

Depending on the location, many species show considerable plasticity in habitat use as juveniles [69]. In our study, juvenile *L. fulviflamma* used mangroves as a nursery area. In Zanzibar, Lugendo [70] showed that juvenile *L. fulviflamma* were found in mangroves but also in sand/mud flats and seagrass areas and were therefore considered as a generalist species. In the Caribbean region, Mateo et al. [67] showed that almost 100% of schoolmaster (*Lutjanus apodus*) collected in mangroves and seagrass beds of St Croix and Porto Rico resided as juvenile in mangrove habitat. This plasticity and variability in effectively used juvenile habitat could be significant enough to limit the general applicability of our results [20]. Nevertheless, our study demonstrates that the spatial distribution of *L. fulviflamma* in New-Caledonia closely match the distribution of its juvenile habitat, which is in agreement with the study by Luiz *et al.* [21] which proposes that post-settlement processes are main determinants of species geographic range. Our study encourages determining which habitats are obligatory or important for juvenile fish in the different regions of the world because these habitats are probably crucial for the spatial distribution of species and would probably require some protection, and/or restoration for species' maintenance and conservation.

## Supporting Information

Figure S1 **Comparison of otolith elemental concentrations between habitats.** Otolith mean value of Ba:Ca, Cr:Ca, Mn:Ca, Rb:Ca, Sn:Ca and Sr:Ca ratios per habitat, (R) reefs and (M) mangroves. Error bars represent standard errors (±SE). Results of one way PERMANOVAs are indicated with (***) p<0.001 and (**) p<0.01.
(TIFF)

Table S1 **Latitudes and longitudes of sampling sites and mean concentrations of elements (in ppm) in the otoliths of** ***L. fulviflamma*** **.**
(XLSX)

Table S2 **Mangrove area, coast length, mean density, biomass and % occurrence of** ***L. fulviflamma*** **in the different lagoons and remote reefs of New Caledonia.**
(XLSX)
