# Supplementary figures and images for: Extent of Mangrove Nursery Habitats Determines the Geographic Distribution of a Coral Reef Fish in a South-Pacific Archipelago
Source: PLoS One. 2014 Aug 20;9(8):e105158. doi: 10.1371/journal.pone.0105158 (PMC4139303; doi:10.1371/journal.pone.0105158)

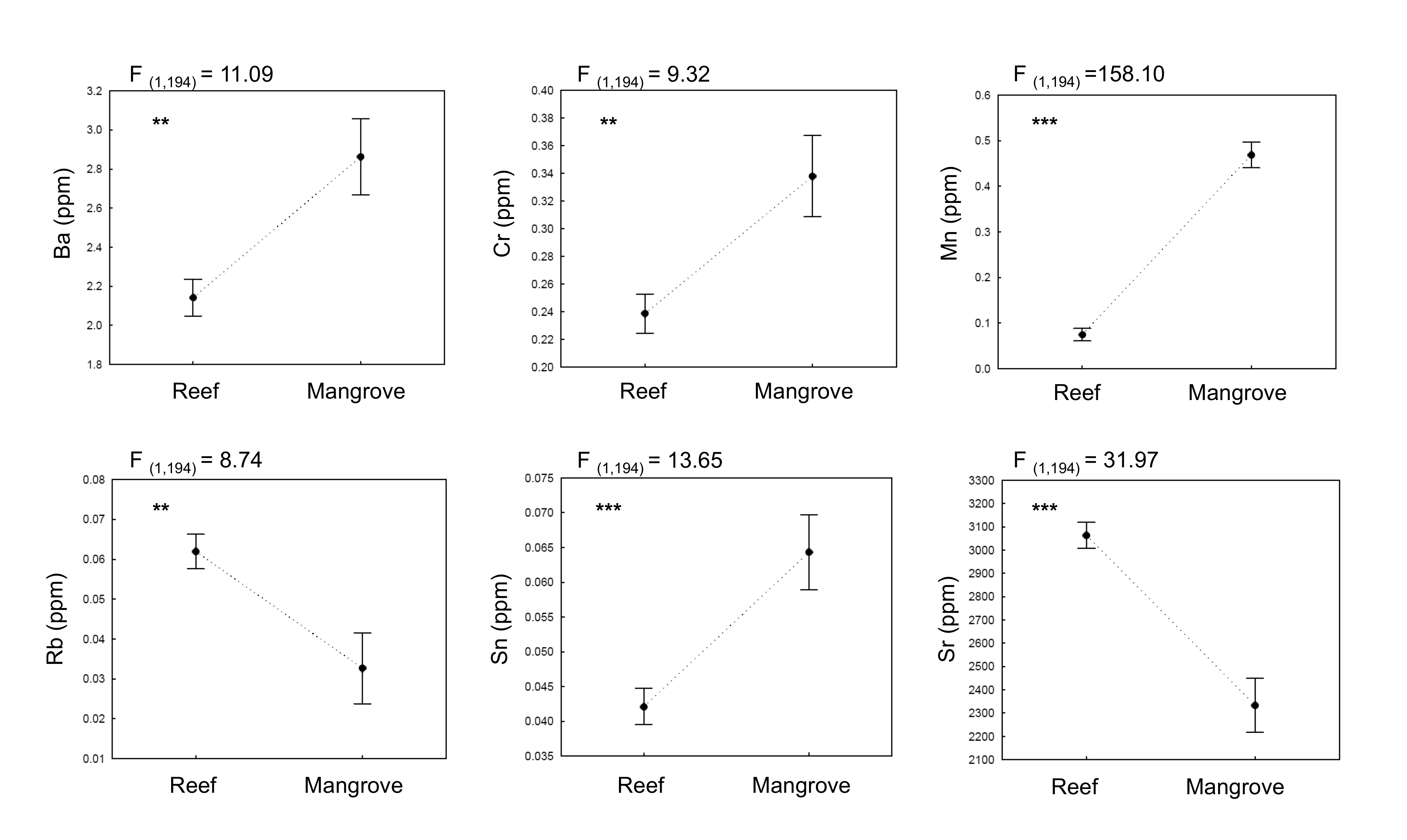

Supplement: Figure S1 — Comparison of otolith elemental concentrations between habitats. Otolith mean value of Ba:Ca, Cr:Ca, Mn:Ca, Rb:Ca, Sn:Ca and Sr:Ca ratios per habitat, (R) reefs and (M) mangroves. Error bars represent standard errors (±SE). Results of one way PERMANOVAs are indicated with (***) p<0.001 and (**) p<0.01. (TIFF) [file pone.0105158.s001.tiff]
